# A 27-Year-Old Female With JAK2 Mutation: A Case of Budd-Chiari Syndrome Secondary to Prolonged Oral Contraceptive Pill Use

**DOI:** 10.7759/cureus.64858

**Published:** 2024-07-18

**Authors:** John P Karns, An Nguyen, Nikita Wong, Aisha True-Malhotra, Dennis Smythe, Raghavendra Vemulapalli

**Affiliations:** 1 Medical School, College of Osteopathic Medicine, Michigan State University, Detroit, USA; 2 Family Medicine, Henry Ford Health System, Detroit, USA; 3 Dermatology, Henry Ford Health System, Detroit, USA; 4 Interventional Radiology, Henry Ford Health System, Detroit, USA

**Keywords:** transjugular intrahepatic portosystemic shunt (tips), jak 2 mutation, multidisciplinary approach, combined oral contraceptive pills, budd-chiari syndrome

## Abstract

Individuals with Philadelphia chromosome-negative myeloproliferative neoplasms (MPNs) such as polycythemia vera and essential thrombocythemia (ET) demonstrate an increased thrombotic risk associated with JAK2 mutations. Physicians must take heed when treating these patients, to mitigate this pro-thrombotic state as much as possible. Failure to do so, or exacerbating the state, can lead to dire consequences. We present the case of a 27-year-old female with a history of ulcerative colitis (UC) and ET, currently taking estrogen-containing oral contraceptive pills (OCPs). She presented to the emergency department with rapid weight gain, jaundice, nausea, and diarrhea and was found to have obstructive jaundice and thrombotic burden that extended into the portal, mesenteric, splenic, and hepatic veins. On the second attempt, a successful transjugular intrahepatic portosystemic shunt procedure was performed, resulting in improved venous flow. This case underscores the importance of cautious medication use, especially OCPs, in patients with hypercoagulable states due to JAK2 mutations, for example, the V617F mutation in JAK2. It emphasizes the need for vigilant monitoring, individualized management, and a multidisciplinary approach to mitigate thrombotic complications. Increased awareness and continued research are crucial for optimizing treatment strategies for patients with MPNs and associated genetic mutations.

## Introduction

Mutations in the JAK gene increase the risk of Philadelphia chromosome-negative myeloproliferative neoplasms (MPNs), which consist of polycythemia vera (PV), essential thrombocythemia (ET), and primary myelofibrosis [1-3].​ The somatic mutation JAK2 V617F, a single nucleotide polymorphism, is present in most patients with PV and MPNs, and in 50% of patients with ET ​[4-7].​ Given the prevalence of PV and ET, which have an incidence between 38 and 57 per 100,000 people in the United States, this is quite significant ​[8].​ In addition, those with PV and ET have a significant burden due to their hypercoagulable state with associated major thrombosis in 20% to 50% at the time of diagnosis ​[9,10].​ This increased hypercoagulable state can lead to the development of portal thrombosis and hypertension, giving rise to Budd-Chiari Syndrome (BCS) [11]. Given these risks, oral contraceptive pills (OCPs) in this population are contraindicated. Our goal is to explain the presentation and treatment of a case of BCS in a 27-year-old female who had combined estrogen-progestin OCP use.

## Case presentation

A 27-year-old female with a history of ulcerative colitis (UC), ET (JAK2 V617F), and generalized anxiety disorder came into the emergency department, referred from an outside hospital, with a chief complaint of weight gain. The patient stated that she gained 9 pounds of weight in the past three days and developed stretch marks due to significant abdominal distention. The patient also reported yellowing of the eyes over the last day and an increase in nausea and diarrhea. The patient was diagnosed with a JAK2 mutation in 2020 after an annual visit revealed thrombocytosis. She closely followed up with a hematologist and was on 81 mg of aspirin daily. The patient's other risk factors for hypercoagulability included a history of well-controlled UC on mesalamine, diagnosed in 2018, and daily use of combined OCPs since 2014. She denied any prior history of thrombosis or anticoagulation use, as well as a family history of malignancy. She did not report any significant alcohol use, intravenous drug use, or recently prescribed medications.

**Figure 1:**
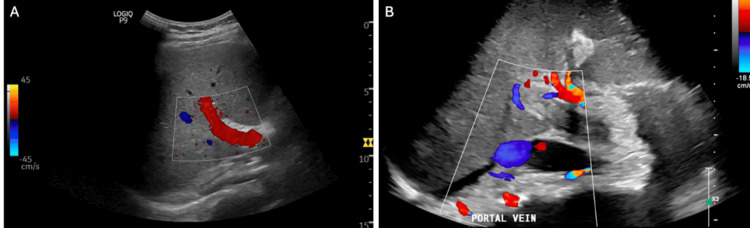
Doppler portal vein ultrasound. (A) Under normal conditions, the portal vein exhibits a monophasic flow pattern on a Doppler ultrasound. This means the blood flow velocity graph has little variation between maximum and minimum velocities, and there is no reversal of flow. (B) The biphasic pattern indicates two distinct phases in the blood flow velocity indicating abnormal pressure dynamics in the portal vein. This blood flow is suggestive of a problem from the liver to the heart.

**Figure 2:**
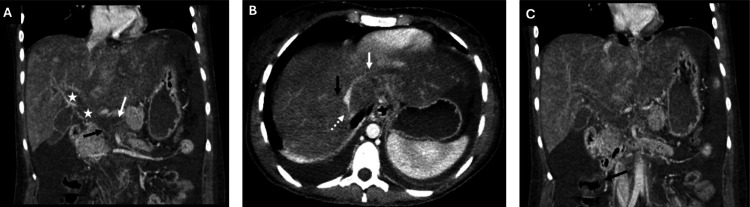
Pre-procedure abdominal CT. (A) Thrombosis of the intrahepatic and extrahepatic portal veins (white star), splenic vein (white arrow), and superior mesenteric vein (black arrow). (B) Thrombosis of the right (dotted white arrow), middle (solid black arrow), and left (solid white arrow) hepatic veins. (C) Bowel wall thickening of the proximal ascending colon (black arrow).

**Figure 3:**
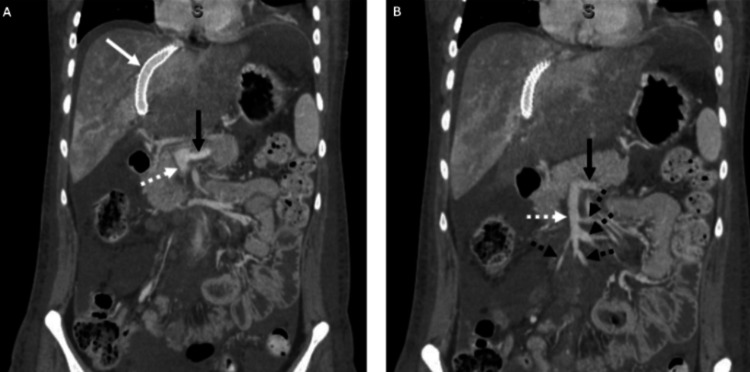
Post-procedure abdominal CT. (A) Coronal CT demonstrating patent TIPS (solid white arrow), patent distal splenic vein (solid black arrow), and superior mesenteric veins (dotted white arrow). (B) Coronal CT demonstrating patent distal splenic vein (solid black arrow), superior mesenteric vein (dotted white arrow), and multiple patent branches of the superior mesenteric vein (dotted black arrow). TIPS, transjugular intrahepatic portosystemic shunt

The initial workup from the outside hospital was significant for obstructive jaundice, and a right upper quadrant ultrasound demonstrated absent portal vein flow, as shown in Figure 1. The patient was transferred to a tertiary care hospital. Upon admission, the patient was noted to have a direct bilirubin of 3.4 and a total bilirubin of 7, indicating an obstructive cholestatic process. The patient also exhibited mild abdominal distention. A CT scan of the chest and liver, as shown in Figure 2, revealed complete thrombosis of the superior mesenteric, splenic, main portal, and intrahepatic portal veins, along with thrombosis of the hepatic veins and indications of colitis in the caudal right colon. It was recommended that the patient undergo a transjugular intrahepatic portosystemic shunt (TIPS) procedure, with options for thrombectomy or thrombolysis by Interventional Radiology (IR). Initially, IR attempted TIPS; however, it was unsuccessful due to the inability to advance wires/catheters into the hepatic veins from an internal jugular approach. Transhepatic access was not attempted per discussion with transplant/hepatobiliary surgery. It was determined that the patient should be evaluated for a multi-visceral transplant, and the workup was initiated inpatient. After the patient was deemed a candidate for multi-visceral transplant and listed, the multidisciplinary transplant team decided to pursue a second attempt at TIPS placement with possible thrombectomy or thrombolysis, intending to qualify the patient for a liver transplant only and avoid the need for a multi-visceral transplant. A repeat CT angiogram of the abdomen and pelvis (CTAP) was obtained before the procedure, showing progression of splenomegaly and partial reconstitution of the right portal vein. The portal vein, splenic vein, and superior mesenteric vein thrombosis appeared similar to previous findings. It also showed a new nonocclusive thrombus within the infrarenal inferior vena cava (IVC) and pulmonary embolism (PE) in the right lower lung. Subsequent CT PE was performed showing multiple right greater than left pulmonary emboli, with no CT evidence of right heart strain. Subsequent bilateral lower extremity (BLE) duplex ultrasound was negative for acute deep vein thrombosis. The TIPS procedure with thrombectomy was then completed, and CT scans were performed before and after, demonstrating a patent TIPS stent and improved flow in the splenic and mesenteric veins, as shown in Figure 3.

## Discussion

The presented case demonstrates the intricate interplay between genetic predispositions, medication use, and the development of severe complications such as BCS. Of particular significance in this case is the prolonged use of OCPs. While OCPs are widely utilized for contraception and management of various gynecological conditions, their thrombotic risk is notably increased in individuals with underlying hypercoagulable states. Though not fully understood, these hypercoagulable states are thought to be from a combination of inhibiting the coagulation cascade, modulating platelets, and affecting the hepatic synthesis of proteins involved in coagulation and fibrosis.​[12]​ These effects would be especially prevalent in those with MPNs and JAK2 mutations [13,14].​ Though limited by its scope, our case report underlines these associations. The decision to prescribe OCPs in such patients warrants careful consideration, given the potential for catastrophic thrombotic events. In addition to OCP use, the clinical presentation of BCS can be nonspecific and variable, posing diagnostic challenges. In this case, the patient's symptoms, including weight gain, abdominal distention, jaundice, and gastrointestinal complaints, prompted further investigation. Imaging studies, particularly CT imaging, played a pivotal role in confirming the diagnosis by revealing the extensive thrombosis involving the hepatic vasculature. With the diagnosis of BCS, the setting of concurrent MPN and JAK2 mutation poses formidable challenges. The decision-making process, including the choice between thrombectomy, thrombolysis, and potential transplantation, requires multidisciplinary collaboration and careful risk-benefit assessment. This includes the goal of restoring flow as soon as possible to mitigate oxidative stress/free radicals and prevent further centrilobular cellular necrosis ​[15].​ Moving forward, this case underscores the importance of heightened awareness among healthcare providers regarding the thrombotic risk associated with JAK2 mutations, particularly in the context of medication use such as OCPs. Furthermore, it highlights the need for individualized management approaches that integrate clinical expertise, patient preferences, and emerging therapeutic modalities. 

## Conclusions

This case serves as a poignant reminder of the heightened thrombotic risk associated with JAK2 mutations, particularly in the context of Philadelphia chromosome-negative MPNs such as PV and ET. 

Careful consideration must be given to the use of certain medications, especially OCPs, in patients with underlying hypercoagulable states. Regular follow-up with a primary care physician and trending of liver function and coagulation studies are imperative to mitigate the risk of catastrophic thrombotic events. 

Moving forward, heightened awareness among healthcare providers regarding the thrombotic risk associated with JAK2 mutations is crucial to prevent similar cases of extensive thromboses, such as BCS presenting in this young woman. This case underscores the need for a multidisciplinary approach, continued research to elucidate optimal management strategies, and the development of novel therapeutic interventions aimed at reducing thrombotic complications in patients with MPNs and associated genetic mutations.
